# Epidemiological Profile and Risk Factors Related to Sexually Transmitted Infections in Sex Workers in Granada (Spain)

**DOI:** 10.3390/nursrep15030082

**Published:** 2025-02-28

**Authors:** Isabel Llavero-Molino, María Sánchez-Torres, César Hueso-Montoro, Alberto González-García, Inmaculada García-García, Francisco Jiménez-Bautista, María Ángeles Pérez-Morente

**Affiliations:** 1 Maternal and Child Hospital, 23007 Jaén, Spain; ilm00014@red.ujaen.es; 2 Virgen de la Arrixaca University Clinical Hospital, El Palmar, 30120 Murcia, Spain; marikillast@hotmail.com; 3 Department of Nursing, Faculty of Health Sciences, University of Jaén, 23700 Jaén, Spain; chueso@ujaen.es; 4 Instituto Biosanitario Granada (IBS. Granada), 18001 Granada, Spain; 5 Centro de Investigación Mente, Cerebro y Comportamiento (CIMCYC), University of Granada, 18071 Granada, Spain; 6 Department of Nursing, Faculty of Health Sciences, University of Granada, 18071 Granada, Spain; igarcia@ugr.es (I.G.-G.); mmorente24@ugr.es (M.Á.P.-M.); 7 Department of Social Anthropology, Faculty of Education Sciences, University of Granada, 18071 Granada, Spain; fjbautis@ugr.es

**Keywords:** sex workers, risk behaviors, sexually transmitted infections, nursing

## Abstract

**Background:** Sex work is one of the oldest trades in the world. It is the practice of sexual activity in exchange for money or material goods. It has traditionally been carried out by women, although in recent years, there has been an increase in the number of male sex workers. Sex workers have been recognized as a population at high risk of contracting and transmitting sexually transmitted infections and human immunodeficiency virus and have had limited access to multiple services, including health care. **Objective:** The aim was to identify the epidemiological profile and risk factors of a population of sex workers in the province of Granada, southern Spain. **Methods:** An analytical cross-sectional study was conducted by reviewing the medical records at a specific sexually transmitted infection center in Spain. **Results:** A total of 157 sex workers’ records were analyzed, most of whom were women, who mainly identified as heterosexual, with a mean age of 28.52 years and a high percentage of foreign nationality. Some sexual behaviors associated with the acquisition of sexually transmitted infections were also analyzed. **Conclusions:** The results revealed a deficient coverage of the health needs of a highly vulnerable and stigmatized social group, highlighting the need for interventions to prevent risky habits, as well as to promote behaviors aimed at achieving better sexual health.

## 1. Introduction

Sex work is one of the oldest known trades throughout human history. It can be defined as the process of offering a sexual act in exchange for money or material goods. Since its inception, the trade has been practiced mostly by women. In recent decades, the term prostitution has been replaced by sex workers (SWs), including women, men, or transgender adults and young people (over 18) [1,2].

It is very difficult to quantify the number of people involved in sex work, as it is a profession carried out in a hidden or clandestine manner in many cases [3]. In Spain, sex work is a particularly complex activity, as it is not a regulated profession. With regard to the legal sphere, it should be noted that Spain is in a situation defined by some authors as “legal limbo”, characterized by neither legalization nor prohibition, leaving the issue of sex work totally undefined, so that its practice is neither legal nor illegal [4].

According to data provided by Médecins du Monde (an international non-profit organization that works with the most vulnerable populations so that they have access to adequate health care), in Spain, during 2023, a total of 25,259 people in sex work accessed its services: 93% cisgender women, 5% transgender women, and 2% cisgender men. It should be noted that of the 7512 women who provided their administrative status, 42% stated that they were in an irregular administrative situation, this being the main reason for entering sex work and also the main barrier to leaving. This situation is also one of the main barriers to access to most of the fundamental rights: housing, health care, and dignified employment [5]. In this regard, data offered by the police and some Non-Governmental Organizations (NGOs) pointed to Spain as one of the countries with the highest consumption of sex work worldwide, with more and more young men going to brothels and paying for sexual services [3].

Both nationally and internationally, SWs have been recognized as a high-risk population for contracting and transmitting sexually transmitted infections (STIs) and human immunodeficiency virus (HIV). STIs constitute a major public health problem worldwide because of the morbidity and mortality associated with them. HIV is highly correlated with STIs as it is aggravated by STIs, and both are sexually transmitted. Globally, it is estimated that SWs are 30 times more likely to be living with HIV than other women of reproductive age. The Joint United Nations Program on HIV/AIDS estimated an average HIV prevalence of 36% in this population. The average reported prevalence of active syphilis among SWs is 10.8%, although less is known about the prevalence or incidence of other STIs and viral hepatitis infections among SWs [6].

Traditionally, SWs have been deprived of many services, including health care. Several studies have pointed out that monitoring and intervention aimed at improving sex workers’ health paradoxically emerged after the possibility of HIV being transmitted to the heterosexual population through SWs became known, which generated moral panic and led to the initiation of strong epidemiological surveillance of SWs [7,8]. However, we must note that currently one of the main groups that contribute significantly to the transmission of HIV are men who have sex with men (MSM). Various epidemiological studies have shown that this group faces a substantially higher risk compared to others, such as SWs, showing higher rates of HIV infection and transmission [9]. Furthermore, the stigmatization of sex work contributes to the alienation of this population from conventional health structures [10]. It follows that health-related interventions for SWs have focused on reducing the risk of transmitting infections to the general population rather than on the proper prevention and treatment of infections.

It should be noted that women are more likely to become infected than men because of their greater biological vulnerability, for example, the lining of the vagina is thinner than the skin of the penis, which makes it easier for viruses and bacteria to penetrate, and the vagina has the ideal conditions for bacterial growth [11]. In addition to biological factors, other factors explain the greater vulnerability of SWs to STIs/HIV, the most relevant being those related to their sexual behavior. Thus, a higher number of sexual partners, inconsistent condom use, early initiation of sexual intercourse, and participation in group sexual practices have been identified as high-risk factors [1,12,13,14,15,16]. In addition to these, numerous studies have documented elevated rates of alcohol and drug use among SWs, which would further increase vulnerability to STIs/HIV [17,18,19,20].

These individual-level risk behaviors are reinforced by structural risk factors such as poverty, stigma, discrimination, language barriers, client violence, immigration, and the criminalization of sex, all of which hinder adequate access to STI/HIV prevention and care services [14,21]. Therefore, the aim of this study was to identify the epidemiological profile and risk factors of a population of sex workers in the province of Granada, southern Spain.

## 2. Materials and Methods

An analytical cross-sectional study was carried out by reviewing the medical records of the Centre for Sexually Transmitted Diseases and Sexual Orientation of the province of Granada during the period 2000–2018. From a sample of 1828 records corresponding to adult subjects without cognitive impairment who attended the center for reasons of suspicion or presence of an STI, a total of 157 records corresponding to subjects who reported working as SWs were analyzed.

The sample size was calculated to detect differences in a binary variable (in this case, the presence or absence of STI), seeking to detect differences of 20% in two years, with a statistical power of 80%, provided that the test was performed with an error of α = 5%. The number of clinical records needed per year was 97. In order to select the records, the first and last record numbers were taken from the archive of each year’s new records. Subsequently, an annual sample was extracted using systematic random sampling [22]. For our study, the subsample of sex professionals within the previously selected sample was analyzed.

The following variables were collected: socio-demographic (age, sex, nationality, employment status, educational level, and sexual orientation); health care (previous consultation, service with whom previous consultation was made); and sexual behavior and clinical variables (regular partner, presence of symptoms in the regular partner, days since last contact without a condom, type of sex work, drug use, previous STIs, age of onset of first sexual intercourse). In addition, sexual behavior was collected according to the classification included in clinical history.

STI diagnosis was considered as a dependent variable and was coded as a dichotomous variable (yes/no). A positive diagnosis was considered when the subject presented symptoms compatible with STIs and there was a positive result in the diagnostic test. If these criteria were not present, the diagnosis was considered negative for STI. This variable was compared with the other variables mentioned above, which were considered independent variables for this analysis.

The data were collected using a form designed specifically for this purpose and subsequently transferred to a computerized database. For quantitative variables, descriptive statistics were calculated, including the mean, median, 95% confidence interval, and interquartile range. In the case of qualitative variables, absolute frequencies and percentages were determined. Subsequently, bivariate analyses were performed to examine the association between the dependent variable and the independent variables, using the Chi-square test (with Fisher correction or likelihood ratio, as appropriate) and the Mann–Whitney U test. The normality of the quantitative variables was evaluated using the Kolmogorov–Smirnov test, ruling out its presence. A statistical significance level of *p* < 0.05 was developed for all analyses, which were performed using IBM SPSS Statistics software, version 28 (SPSS, Inc., Chicago, IL, USA).

The biomedical research ethics committee of the province of Granada (PEIBA) approved this study on 7 March 2013 for data collection from 2000 to 2012 (code No. 1766-N-18). Subsequently, the same ethics committee approved the extension of data collection to the period 2013–2014 on 1 April 2015. Finally, the same ethics committee approved the last extension of the collected data to the period 2015–2018 on 19 January 2019. Therefore, the data were collected at three different points in time: in April 2013 (first data collection period); in May 2015 (second data collection period); and in February 2019 (third data collection period). All data were treated with the utmost confidentiality and in compliance with Organic Law 15/1999, of 13 December, on the Protection of Personal Data, and Organic Law 3/2018, of 5 December, on the Protection of Personal Data and guarantee of digital rights.

## 3. Results

The final sample consisted of 157 medical records corresponding to self-identified SWs, of whom 5.7% were male and 94.3% were female. Of these, 94.8% identified themselves as heterosexual, 2% as bisexual, and 3.3% as homosexual. The mean age (n = 157) was 28.52 years (95% CI = 27.45–29.60; median = 28; interquartile range = 23–33), and 5.8% had completed higher education. The data revealed that 89.9% of the sample were foreigners.

Approximately half of the subjects, 56.6%, reported having a regular partner, and of these, 53.2% provided data about their partner’s symptomatology. More than three-quarters of the subjects, 75.8%, carried out sex work in clubs, followed by 20.8% who reported sex work in flats, and 1.7% on the street. Illegal drug use was reported to be low, with 87.7% stating that they did not use illegal drugs, compared to 34.4% who reported using illegal drugs. The mean age at first sexual intercourse (n = 109) was 16.81 years (95% CI = 16.38–17.24; median = 17; interquartile range = 15–18).

In relation to the variable “sexual behaviors”, there was a slight predominance of anal sex, which was reported by 54.14%, followed by vaginal sex reported by 50.9%, and oral sex by 47.13%. Condom use was low for oral sex, being declared by 68 subjects, of whom only 26.5% always use it; for anal sex, its use was declared by 29 subjects, of whom 55.2% indicated using it in all their relations; and in the case of vaginal sex, 74% who declared this practice stated that they used it in all their practices. Table 1 shows the socio-demographic variables, sexual behaviors, and clinical variables of the study sample.

STI diagnosis was recorded in 92 cases, with a negative diagnosis in 53 (57.6%) and a positive diagnosis in 39 (42.3%). In records with a positive diagnosis, the mean age (n = 39) was 27.31 years (95% CI = 25.06–29.55; median = 24; interquartile range = 23–34); in records with a negative diagnosis, the mean age (n = 53) was 28.40 years (95% CI = 26.99–29.80; median = 28; interquartile range = 24–33). There were no statistically significant differences (*p* = 0.167).

When comparing STI diagnosis with the rest of the independent variables, statistically significant differences were found in the nationality, sexual orientation, and age at first sexual intercourse variables, with no significant differences in the rest of the variables (Table 2). In records with a positive diagnosis, the mean age at first sexual intercourse (n = 39) was 15.97 years (95% CI = 15.15–16.78; median = 16; interquartile range = 14–17); in records with a negative diagnosis, the mean age at first sexual intercourse (n = 53) was 17.33 years (95% CI = 16.55–18.16; median = 17; interquartile range = 16–18). There were statistically significant differences (*p* = 0.006).

As noted, only 39 cases obtained a positive diagnosis of sexually transmitted infections. A total of 22.8% of the cases obtained a diagnosis of human papillomavirus, followed by *Treponema pallidum* with 8.6%, *Chlamydia trachomatis* with 5.4%, and HIV with 4.3%. These data are shown in Table 3.

## 4. Discussion

The present study provides data to contribute to a deeper understanding of the socio-demographic characteristics and risk indicators in the epidemiology of STIs in SWs attending a specialized referral center for STI care.

The mean age of the subjects analyzed was 28.52 years. This is a high mean compared to other studies reviewed, where the mean age was 18.2 and 22.6 years, respectively [13,23]. However, we also found studies where the mean age is close to that of our study at 26.5 [24] and 27.7 years [25]. The variability in the mean age can be explained by the socio-demographic differences in the samples analyzed in each of the studies due to the difficulty of access and participation of this population group. Probably the onset of activity as a SW begins at earlier ages than those found in our study; however, limitations in access to the health system, stigma, and low socio-educational conditions associated with a lower knowledge of available resources may delay demand and general access to the health system.

The majority of the subjects in our study were women. This gender difference shows how socio-cultural patterns that associate sex work with women continue to be perpetuated today. The literature also indicates that men are less willing to consult health professionals, a situation that is aggravated in vulnerable social groups such as SWs and with stigmatized diseases such as STIs and HIV [26]. Sexual orientation, mostly heterosexual, showed statistical significance with respect to the presence of STIs; however, in other similar studies analyzed, this variable was not included [12,15,16,25].

With regard to nationality, it is noteworthy that the majority of the population studied was of foreign origin. Several studies have documented that SWs who have migrated are at higher risk of contracting STIs and HIV than those who have not migrated. The reasons for the risk among more mobile populations are attributed to socio-economic characteristics and difficulties in accessing health care and other social services. The reasons for mobility were cited as the search for work and an escape from the social stigma of family and community members. In addition, the results indicated that highly mobile SWs reported more inconsistent condom use than those with low mobility [10,27]. A new environment, competition to acquire clients, lack of social support or protective structures, and high economic need are some of the contextual factors that appear to drive inconsistent condom use by mobile SWs.

The educational attainment of the population was low, with only 5.8% of SWs having tertiary education. In our opinion, the low levels of education in general contribute in multiple ways to increased vulnerability to STIs through fewer resources available for negotiating the use of protective methods, lack of knowledge, and self-perceived risk of STIs and HIV. Schooling is one of the most important factors in measuring socio-economic status and its effects on the health status of a population [28]. The results of Oliveira and Fernandes [8] indicated that women with lower levels of education were more likely to report having unprotected sex compared to SWs with higher levels of education [13].

In terms of health care indicators, approximately two-thirds of the subjects had not had any previous consultations, and of the 20 who reported having had a previous consultation about their health status, only 5 went to specialized centers. These data highlight, on the one hand, the low attendance to health services by these professionals with the consequent risk of contagion and transmission of STIs and, on the other hand, the important role of specialized STI centers as reference centers for addressing this health problem in specific populations.

Among the sexual behaviors reported, anal sex was the most common, followed by vaginal sex and the mouth-to-penis habit. Condom use was inconsistent across all habits, with condom use being higher in vaginal sex, where 74% said they always used condoms. Special mention should be made of anal sex, which is well documented in the literature as one of the highest risks of sexual activity for contracting infections due to the characteristics of the rectum, which is a delicate tissue that can be easily damaged and is aggravated by limited lubrication [28].

We found that most of the subjects reported carrying out their activity in clubs, as these places were safer for practicing sex work rather than on the streets, in flats, etc. This is consistent with previously published findings in relation to workplaces, where it is shown that SWs in high-risk commercial places (mainly the street) were linked with characteristics of vulnerability that include old age, illiteracy, experiences of violence, low decision-making in the use of condoms, and clients paying them less [29,30]. Robertson et al. [31] noted in their study that clubs favored the creation of regular clients; SWs end up developing closer emotional ties, and it also offers more stable forms of financial support. These types of clients imply a lower risk because instead of frequenting multiple SWs, they are usually “monogamous” and tend to have a single sex-worker partner, as well as a stable partner [30]. It is probable that the socio-economic vulnerability of a woman influences the type of sex work that she engages in and that some sex work environments bear a greater risk of infection than others. As Saggurti et al. [27] pointed out, the most vulnerable women end up devoting themselves to sex work in more insecure environments, which in turn aggravates their vulnerability [29]. A possible explanation is their economic needs, since many clients offer to pay more money for sex without protection. A qualitative study realized in China revealed that a SW was earning double the income when they stopping using condoms [25].

Other findings of this study are in line with previous results, including work risk factors that increase the vulnerability to STIs/HIV of the SWs such as the weak use of condoms and the age of beginning to have sexual intercourse. Understanding these behavioral risk factors is particularly important since substantial evidence exists on the interactive relation between these factors, for example, the use of illegal drugs increases the weak use of condoms among SWs.

In agreement with the findings published previously by other studies on sex workers, our study found a weak use of condoms with clients. The aptitude to negotiate surer sexual practices, which the use of condoms is, has been shown to be a fundamental component of the prevention of STIs/HIV [31]. Benoit et al. [32] demonstrated that promoting the empowerment of sex workers at the individual and community levels could improve, to a great extent, their negotiation skills for safe sexual intercourse, helping to improve the outcomes of sexual and reproductive health [32].

More than half of the SWs in our study (56.6%) were part of a habitual couple, which was understood as one with whom regular sexual intercourse is had without economic compensation. Our study does not contribute specific information on the use of condoms with a habitual partner; nevertheless, diverse studies reveal the use to be very low in this type of relationship. All the studies that examined the use of condoms by sex workers stratified by the type of sexual couple (who pay or do not pay) indicated a significantly lower use valuation with couples who do not pay [14]. Robertson [31] pointed out that the mutual confidence and the affection inside the couple were the main reasons why sex workers were not using condoms with habitual partners; additionally, they thought that the use of condoms reduced the intimacy and that it might be perceived by couples as a sign of suspicion and infidelity in the marriage. This fact might be associated with a lower perception of the risk in the context of non-professional relations, which in turn implies a high STI/HIV risk associated with this population bridge; according to Scambler and Scambler [7]: “the sexual work places the clients and the couples of the workpeople of the sex in high infection risk, being able to act like bridge for the illness in the general population” [24]. On the other hand, this indicator suggests the need to extend the field of intervention regarding the prevention and control of STIs to the relationship partners of sex workers [10].

The results of our study showed a lower proportion of SWs who do not consume illegal drugs, as opposed to those who declared themselves to be a consumer, without a significant association being observed between the consumption of illegal drugs and the presence of STIs. This could be due to the inadequate statistical power, given the limited size of the sample and bearing in mind that this variable was only answered by 96 subjects, which might indicate an undervaluation of the SWs who really consume illegal drugs. However, the evidence indicates a high valuation of the consumption of alcohol/illegal drugs prior to or during sexual intercourse [24]. Among the motives for the consumption is a way to face the stress and the violence associated with sex work. Illegal drug consumption affects the judgment of SWs and the ability to negotiate safe sex with clients, driving up the risk of transmission of STIs/HIV between the SW and their clients [23,31]. Considering the significant association between the consumption of illegal drugs and the incidence of STIs/HIV, future studies should explore the dynamics of the consumption of alcohol among SWs.

The age of initiation of sexual intercourse also reached statistical significance, suggesting that SWs initiating sexual practices at an earlier age have a longer period of exposure to sex work and the risks inherent therein and, consequently, higher rates of STIs. These results are consistent with SW research in other contexts [14,20]. However, there are discrepancies, and some authors point to an increased risk in the younger population associated with a lower perception and awareness of risks, leading to involvement in risky sexual acts and other factors that impede preventive practices against STIs/HIV [23].

In reference to the STIs diagnosed in the present sample, only 39 subjects tested positive for one or more STIs, with human papillomavirus, *Treponema pallidum*, *Chlamydia trachomatis*, and *Gardnerella vaginalis* being the predominant infections. In a study conducted by Jacob Lindman [33] in Guinea, the most common STIs were *Chlamydia trachomatis* (11.8%), *Neisseria gonorrhoeae* (10.1%), *Mycoplasma genitalium* (21.9%), *Trichomonas vaginalis* (26.3%), and *Treponema pallidum* (2.8%). The data are consistent with those obtained in our sample but show discrepancies that may be due to the inherent characteristics of the place of origin. On the other hand, in Spain, Laia Ferrer [34] reported in her study that the most prevalent STI was *Neisseria gonorrhoeae* (19.2%), followed by *Chlamydia trachomatis* (10.3%). Another study in Spain reported a prevalence of 1.8% and 0.5% for *Chlamydia trachomatis* and *Neisseria gonorrhoeae*, respectively [35]. These results are consistent with the findings of our study.

## 5. Limitations

Although the findings of this study indicate important implications based on empirical evidence on the relationship between socio-demographic characteristics and risk indicators in the acquisition of STIs in SWs, they should be interpreted with caution in light of several limitations of this study. First, there is the number of subjects analyzed. In order to carry out more robust statistical analyses, it would be necessary to carry out studies with larger populations.

The second limitation is the significant loss of data observed in this study, which can be attributed to the inherent characteristics of research based on the analysis of medical records. In particular, since this is a population with risky sexual behaviors, there are specific factors that make it difficult to collect complete and accurate data. This population faces high social vulnerability and marked stigmas, which contribute to their avoidance of seeking health care services on a regular basis. Even when they access these services, it is common for them not to reveal all the information requested, either out of fear of being judged, distrust of registration systems, or a desire to protect their privacy. In our study, the majority of the data were self-reported through a face-to-face interview, raising concerns about recall bias and social desirability, particularly with sexual behaviors, which may result in underestimations of certain risky behaviors. Due to the significant amount of missing data in the present study, multiple regression analyses were not performed. This type of analysis requires complete data for all variables included in the model, as traditional regression methods are not able to handle missing values efficiently. Given that some variables have a high number of missing cases, the implementation of such analyses would have generated models with very low statistical power and representativeness, which would have compromised the validity of the results.

Thirdly, the possible effect of sampling bias cannot be ruled out since the study sample was made up of SWs who came to the STI center in Granada, leaving out SWs who did not go to the health services center. The sample of sex workers constitutes a subsample of a general sample, which could be a limitation. Fourthly, the cross-sectional nature of this study did not allow causal relationships between risk behaviors and the presence of STIs; it was only possible to establish causal hypotheses. Finally, it is worth noting that the impact of the COVID-19 pandemic, specifically the movement restrictions as part of the social and public health measures against the pandemic, was associated with a limitation in data collection, preventing the collection of these data from 2019 onwards. After the pandemic, it would be interesting to collect data from these years to analyze the impact of the pandemic on STIs in this at-risk population.

## 6. Implications for Clinical Practice

Research on sexually transmitted infections in vulnerable populations such as SWs is crucial for clinical nursing practice. This group faces multiple barriers that limit adequate access to health services, including stigmatization, fear of discrimination, and a lack of inclusive health care policies. All of these difficulties contribute to SWs avoiding seeking health care services on a regular basis. Traditionally, research on SWs has focused on reducing the risk of transmission of infections to the general population; however, a new approach aimed at the prevention and appropriate treatment of infections is needed.

As health professionals, nurses have a key role in the prevention, early detection, and treatment of STIs in these vulnerable populations, contributing to improving public health and the quality of life of these individuals.

This research will allow the development of evidence-based knowledge that will strengthen care strategies and allow the design of care models adapted to the specific needs of this population, ensuring person-centered care based on respect for their rights, promoting equity in access to health, and reducing the burden of these infections in a historically marginalized group.

## 7. Conclusions

We can conclude that the profile of the SWs who came to the STI consultation was mostly young women, of foreign nationality, and self-declared mostly heterosexual. Most reported sexual practices were anal, vaginal, and oral sex, showing inconsistent condom use in all cases. Most of the professionals declared that they carried out their activities in clubs, followed by flats and the street. Regarding the consumption of harmful substances, our study shows a low consumption. The only statistically significant differences were found in the STI diagnoses when considering the nationality, sexual orientation, and age of first sexual intercourse variables, and no significant differences were found in any of the other variables.

The results highlight the poor coverage of health needs in a highly vulnerable and stigmatized group such as SWs and highlight the urgent need for interventions to prevent risky sexual practices such as inconsistent and incorrect condom use in SWs, as well as STIs/HIV risk perception and safe sex negotiation skills.

Since most STIs are asymptomatic, their detection depends to a large extent on screening and treatment coverage of the most vulnerable populations, including those with a greater number of sexual partners and those whose practices are associated with an increased risk of acquisition and transmission. When it comes to sex work, factors arise that pose additional challenges to the diagnosis, intervention, and control of STIs, such as the fact that sex work is carried out mostly by mobile (foreign) women.

## Figures and Tables

**Table 1:** Socio-demographic variables, sexual behaviors, and clinical variables of the study sample.

		n	%
Sex (n = 157)	Man	9	5.7%
	Woman	148	94.3%
Nationality (n = 157)	Spanish	16	10.2%
	Foreigner	141	89.8%
Employment situation (n = 145)	Active	140	96.6%
	Unemployed	5	3.4%
Level of instruction (n = 139)	Higher Education	8	5.8%
	The rest	131	94.2%
Sexual orientation (n = 153)	Heterosexual	145	94.8%
	Bisexual	3	2.0%
	Homosexual	5	3.3%
Prior consultation (n = 94)	Yes	20	21.3%
	No	74	78.7%
Regular couple (n = 136)	Yes	77	56.6%
	No	59	43.4%
Usual partner has symptoms (n = 41)	Yes	5	12.2%
	No	36	87.8%
Last days since contact without condom (n = 95)	Never	13	13.7%
	Less than 1 month	44	46.3%
	1 to 6 months	29	30.5%
	6 months or more	9	9.5%
Type of sex work (n = 120)	Club	91	75.8%
	Apartment	25	20.8%
	Other	4	3.3%
Drug use (n = 96)	Yes	33	34.4%
	No	63	65.6%
Previous STIs (n = 122)	Yes	33	27.0%
	No	89	73.0%
Vaginal sexual behavior (n = 80)	Never/Sporadically	0	0%
	Often/Always	80	100%
Condom use vaginal sex (n = 77)	Never/Sporadically	7	9.1%
	Often/Always	70	90.9%
Oral sexual behavior (n = 74)	Never/Sporadically	1	1.4%
	Often/Always	73	98.6%
Condom use oral sex (n = 68)	Never/Sporadically	28	41.2%
	Often/Always	40	58.8%
Anal sexual behavior (n = 85)	Never/Sporadically	66	77.6%
	Often/Always	19	22.4%
Condom use anal sex (n = 29)	Never/Sporadically	6	20.7%
	Often/Always	23	79.3%

Abbreviation: STIs = Sexually transmitted infections.

**Table 2:** Socio-demographic characteristics and sexual behaviors vs. STI diagnosis.

		No STIs		Yes STIs		
		n	%	n	%	*p*
Sex (n = 92)	Male	1	1.9%	5	12.8%	0.080
	Female	52	98.1%	34	87.2%	
Nationality (n = 92)	Spanish	2	3.8%	7	17.9%	0.033 *
	Foreigner	51	96.2%	32	82.1%	
Employment situation (n = 88)	Active	50	100%	36	94.7%	0.184
	Unemployed	0	0%	2	5.3%	
Level of Instruction (n = 85)	Superiors	3	6%	1	2.9%	0.640
	All others	47	94%	34	97.1%	
Marital status (n = 90)	Single	42	80.8%	33	86.8%	0.445
	All others	10	19.2%	5	13.2%	
Sexual orientation (n = 89)	Heterosexual	51	100%	33	86.8%	0.012 *
	Bisexual/Homosexual	0	0%	5	13.2%	
Prior consultation (n = 56)	Yes	6	20.7%	8	29.6%	0.440
	No	23	79.3%	19	70.4%	
Has a regular partner (n= 84)	Yes	32	64.0%	20	58.8%	0.632
	No	18	36.0%	14	41.2%	
Usual partner has symptoms (n = 26)	Yes	1	6.3%	2	20.0%	0.538
	No	15	93.7%	8	80.0%	
Days since last contact without a condom (n = 59)	Never	8	25%	2	7.4%	0.331
	Less than 1 month	13	40.6%	13	48.1%	
	Between 1 and 6 months	9	28.1%	10	37.0%	
	6 months or more	2	6.3%	2	7.4%	
Type of sex work (n= 69)	Club	32	76.2%	17	63%	0.474
	Apartment	8	19.0%	9	33.3%	
	Other	2	4.8%	1	3.7%	
Illegal drug use (n = 52)	Yes	10	33.3%	10	45.5%	0.403
	No	20	66.7%	12	54.5%	
Previous STIs (n= 72)	No	35	81.4%	19	65.5%	0.127
	Yes	8	18.6%	10	34.5%	
Vaginal sexual behavior (n = 45)	Frequently	5	22.7%	2	8.7%	0.189
	Always	17	77.3%	21	91.3%	
Condom use vaginal sex (n = 43)	Never/Sporadically	2	10%	2	8.7%	0.435
	Frequently/Always	18	90%	21	91.3%	
Oral sexual behavior (n = 41)	Never	0	0%	1	4.2%	0.498
	Frequently/Always	17	100%	23	95.8%	
Condom use oral sex (n = 38)	Never/Sporadically	6	37.5%	9	40.9%	0.705
	Frequently/Always	10	62.5%	13	59.1%	
Anal sexual behavior (n = 51)	Never/Sporadically	19	82.6%	20	71.4%	0.534
	Frequently/Always	4	17.4%	8	28.6%	
Condom use anal sex (n = 19)	Never	0	0%	3	21.4%	0.219
	Frequently/Always	5	100%	11	78.6%	

Abbreviations: STIs = Sexual transmitted infections. * = Statistically significant, *p* < 0.05.

**Table 3:** STIs diagnosed.

STI	n	%	95% CI
Human papillomavirus	21	22.8	15.4–32.3
*Treponema pallidum*	8	8.6	4.4–16.2
*Chlamydia trachomatis*	5	5.4	2.3–12.1
*Gardnerella vaginalis*	5	5.4	2.3–12.1
Human immunodeficiency virus	4	4.3	1.7–10.6
Herpes simplex	2	2.1	0.6–7.5
*Trichomonas vaginalis*	2	2.1	0.6–7.5
*Neisseria gonorrhoeae*	1	1	0.19–5.9
Hepatitis B virus	1	1	0.19–5.9
Hepatitis C virus	1	1	0.19–5.9
*Molluscum contagiosum*	1	1	0.19–5.9
*Mycoplasma genitalium*	1	1	0.19–5.9

Abbreviations: STIs = Sexual transmitted infections. CI = Confidence interval.

## Data Availability

Data sets generated during the current study are available from the corresponding author on reasonable request.

## Public Involvement Statement

No public involvement in any aspect of this research.

## Guidelines and Standards Statement

This manuscript was drafted against the Strobe guide.
